# A bibliometric analysis of hotpots and trends for the relationship between skin inflammation and regeneration

**DOI:** 10.3389/fsurg.2023.1180624

**Published:** 2023-04-21

**Authors:** Zhen-jiang Liu, Mei-juan Wang, Jia Luo, Ya-ting Tan, Min Hou, Shu-chao Wang

**Affiliations:** ^1^ Department of Cardiology, Cardiac Catheterization Lab, The Second Xiangya Hospital of Central South University, Changsha, China; ^2^ Medical Imaging Center, Qingdao West Coast New District People's Hospital, Qingdao, China; ^3^ Hunan key Laboratory of the Research and Development of Novel Pharmaceutical Preparations, Changsha Medical University, Changsha, China; ^4^ Center for Medical Research, The Second Xiangya Hospital of Central South University, Changsha, China; ^5^ Clinical Nursing Teaching and Research Section, The Second Xiangya Hospital of Central South University, Changsha, China; ^6^ Party Committee Office, The Second Xiangya Hospital of Central South University, Changsha, China; ^7^ National Clinical Research Center for Mental Disorders, The Second Xiangya Hospital of Central South University, Changsha, China

**Keywords:** bibliometric, Citespace, VOSviewer, wound, skin regeneration, inflammation, immune

## Abstract

**Background:**

Skin regeneration is a challenging issue worldwide. Increasing research has highlighted the role of immune cells in healing and the underlying regulatory mechanism. The purpose of this study was to identify the hotspots and trends in skin regeneration and inflammation research through bibliometrics and to provide insights into the future development of fundamental research and disease treatment.

**Methods:**

Publications were collected from the Web of Science Core Collection on March 1, 2022. Articles and reviews published in English from January 1, 1999, to December 31, 2022, were selected, and statistical analyses of countries, institutions, authors, references, and keywords were performed using VOSviewer 1.6.18 and CiteSpace 5.8.

**Results:**

A total of 3,894 articles and reviews were selected. The number of publications on skin inflammation and regeneration showed an increasing trend over time. Additionally, authors and institutions in the United States, United Kingdom, Canada, and China appeared to be at the forefront of research in the field of skin inflammation and regeneration. Werner Sabine published some of the most cited papers. *Wound Repair and Regeneration* was the most productive journal, while *Journal of Investigative Dermatology* was the most cited journal. Angiogenesis, diamonds, collagen, cytokine, and keratinocytes were the five most commonly used keywords.

**Conclusion:**

The number of publications on skin inflammation and regeneration show an increasing trend. Moreover, a series of advanced technologies and treatments for skin regeneration, such as exosomes, hydrogels, and wound dressings, are emerging, which will provide precise information for the treatment of skin wounds. This study can enhance our understanding of current hotspots and future trends in skin inflammation and regeneration research, as well as provide guidelines for fundamental research and clinical treatment.

## Introduction

As the largest organ, the skin plays an essential role in protecting the body from various threats, such as trauma, noxious agents, UV radiation, and pathogens (1, 2). When the skin is injured, localized injury can lead to persistent damage, compromising its protective function and disturbing the wound-healing process (3, 4). Skin regeneration is a challenging issue worldwide and comprises three main stages: inflammation, re-epithelialization, and skin remodeling (5, 6). This process involves a series of cell types, such as endothelial cells, fibroblasts, stem cells, and immune cells (5, 7). Traditionally, immune cells are thought to cause systemic damage and destruction (8–10). In 1968, Elie Metchnikoff first mentioned the presence of immune cells at the site of injury (11). Since then, researchers have discovered that immune cells not only eliminate microbial and cellular debris but also promote tissue growth and wound repair (12, 13). Furthermore, increasing research has revealed the crucial role of immune cells in healing as well as the underlying regulatory mechanism (14, 15). In other clinical studies, immunocompromised individuals have been reported to exhibit impaired wound repair, which also indicates the important role of immune cells in skin healing (16–18). Currently, increasing evidence indicates that skin regeneration is not an autonomous process but one that relies on the regulation of inflammation. Investigating the hotspots and trends in this field can help researchers understand the fundamental, latest, and most influential advances as well as highlight potential research directions.

There are two types of studies that can summarize related publications in a specific research direction: reviews and bibliometrics (19, 20). Bibliometrics may serve as a useful summary and predictive tool for new researchers looking to explore a research field and expand their research horizons (21, 22). Bibliometric analysis reflects current research hotspots and potential research directions (23) and is a discipline that uses mathematical and statistical methods to study and quantify the contribution of publications to a specific field of research (22). It can also help organize large volumes of unstructured data into a rigorous, meaningful, and scientific arrangement (24). Therefore, bibliometric studies help build a strong base for researchers in specific areas. Two software, CiteSpace and VOSviewer, are commonly used for visual analysis (25). These software provide researchers a comprehensive insight into the field of research (26), which then enables them to assess progress in both qualitative and quantitative research and position their anticipated contributions to specific areas of research (26).

In this study, we aimed to provide a comprehensive overview of the relationship between skin regeneration and inflammation using bibliometrics. Furthermore, we summarize the number of publications, citation frequency, and centrality to analyze current research hotpots and other research directions. Finally, we review the latest research results, technology, and treatment for skin regeneration. This research can help new researchers select appropriate research directions and provide basic knowledge and understanding of current research. Furthermore, it could provide guidelines for fundamental research and precise clinical strategies for skin regeneration.

## Data and methods

### Data strategy and selection criteria

**Figure 1:**
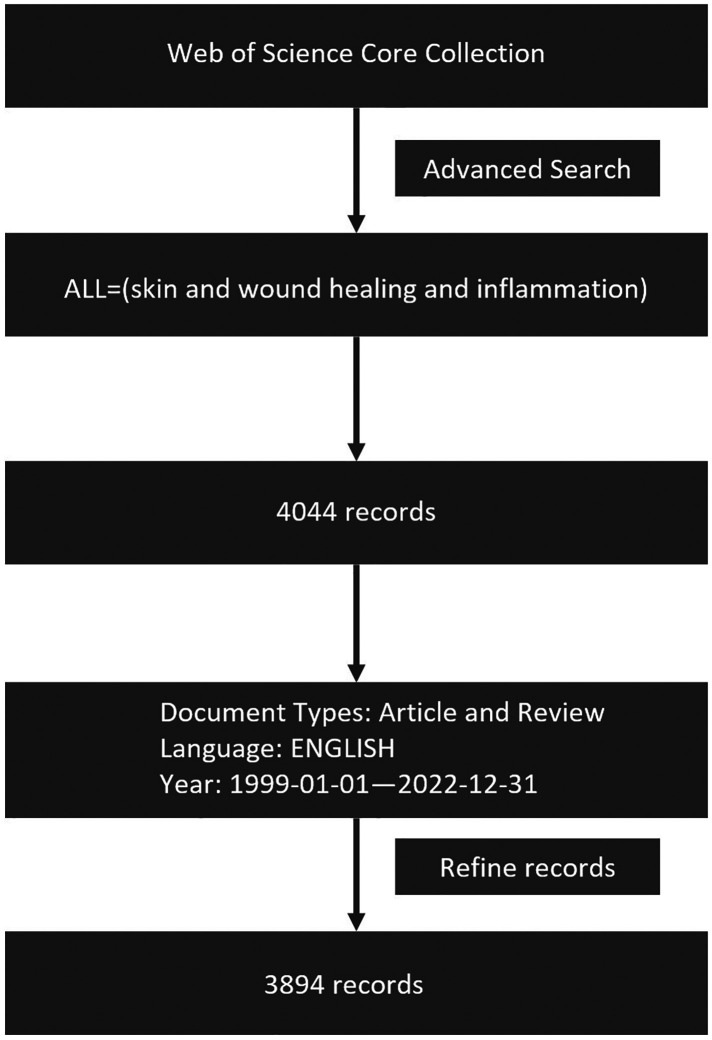
The data collection and retrieval strategy.

The relevant keywords were first searched in the MeSH Database (https://www.NCBI.nlm.nih.gov/mesh). The search formula: ALL = (skin and wound healing and inflammation) was used to search the Web of Science Core Collection. After that, we screened target papers based on (1) Document type: “Article” and “Review,” excluded books, corrections, letters, conference materials, and retracted papers; (2) Publication date: January 1, 1999, to December 31, 2022; (3) Language: English. This work was completed on March 1, 2023. Finally, 3,894 relevant documents met the criteria. Documents were exported as all records and references, and saved as plain text files (.txt) for bibliometric and visual analysis (Figure 1).

### Methodology

The dimensions of this analysis include publication year, author, institution, country and region, journal, keywords, and key references. We downloaded all data imported from the Web of Science (WOS) Core Collection into VOSviewer (version 1.6.18; https://www.vosviewer.com/downloavosviewer) and CiteSpace (version 5.8.R3; https://sourceforge.net/project/citespace/files/latest/do) to perform analyzing and visualizing.

CiteSpace and VOSviewer are the main software that can be used to analyze the scientific knowledge contained in complex data, and display the structure, distribution, and discipline of information through visual methods. Co-occurrence analysis is one method to show the frequency of occurrence of same sentences in one paper to determine their similarities. Therefore, researchers could get to know the research hotspots and future trends. Moreover, co-citation analysis could provide researchers with the first and basic published paper and related knowledge in this specific research area (27). In addition, we used Microsoft Office Excel to analyze the research trends in published papers in the type of tables.

In the figures presented by the visualization map below, each circle represents a node, and the diameter size of the circle represents the frequency of the item that appears in the co-occurrence analysis. The color of a circle is determined by the cluster of categories to which it belongs. The connection between nodes represents the association of the corresponding node, and the strength of the association between nodes is expressed in line width.

## Results

### Annual global publication outputs

**Figure 2:**
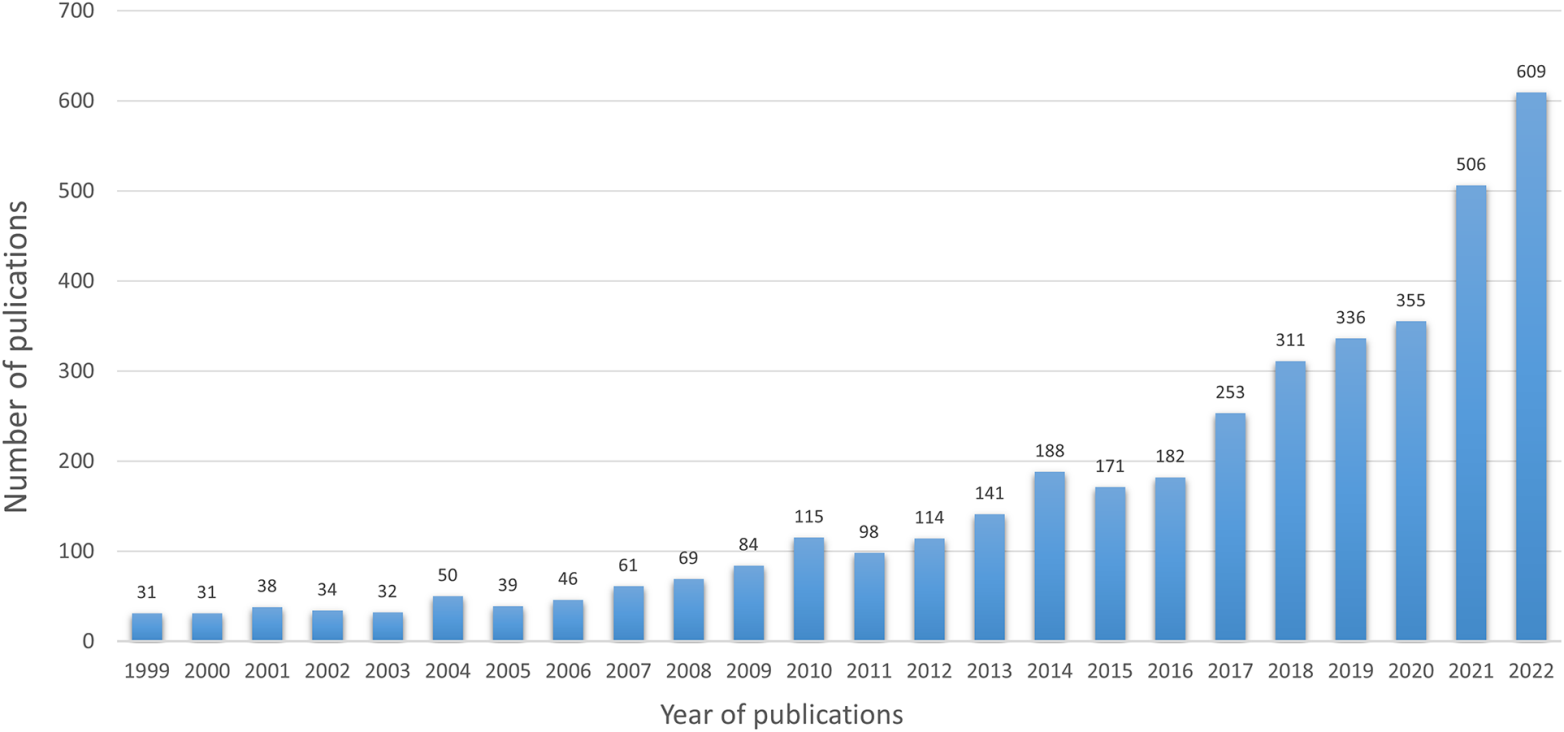
The number of articles per year from 1999 to 2022. The number of literature studies published has grown steadily and peaked in 2022.

The first paper, according to the formula, was published in 1999, and we set the statistical period from 1999 to 2022. The annual publication trends are shown in Figure 2. The number of papers increased from 1999 (*n* = 31) to 2022 (*n* = 609). The increase in the number of published papers over the last 2 years indicates that research on skin inflammation and regeneration has received considerable attention.

### Co-authorship of countries/regions

**Figure 3:**
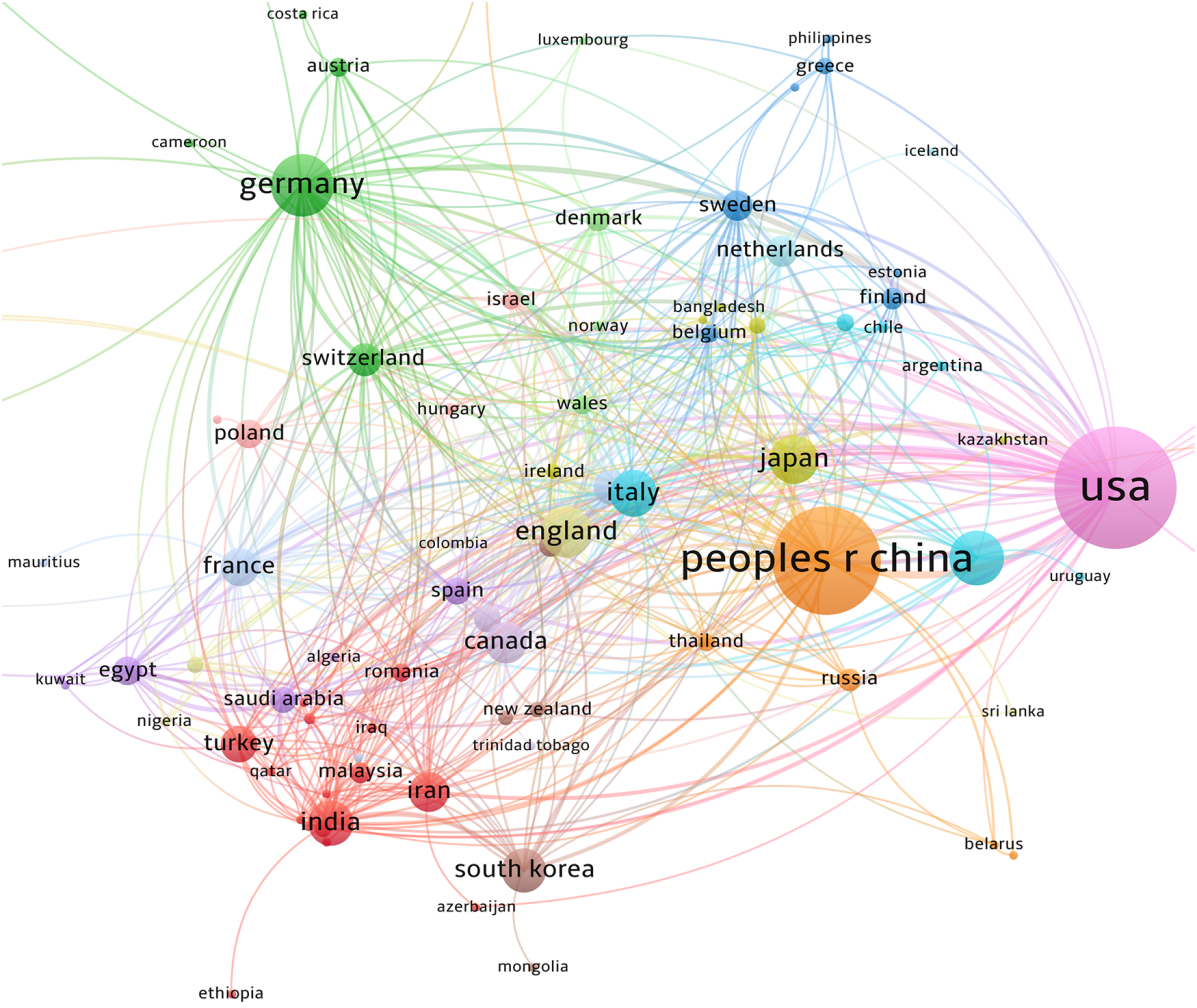
The co-authorship network of countries/regions. A total of 99 countries/regions have participated in the study. China and the United States are the centers of the study. The largest cluster is centered on the United States. China's cooperation between countries and regions is relatively strong.

Research studies conducted in 99 countries/regions were collected in this study. Figure 3 shows the cooperation between countries/regions. Table 1 lists the top 10 countries with the highest number of publications. The United States (1,089 publications, 57,054 citations) is the most productive country, followed by China (860 publications, 18,269 citations). The United States published the highest number of papers and had the largest number of citations among all countries. Forty-six countries/regions with over 10 papers were divided into six color-coded groups. The largest group consists of 14 countries, focused on the United States, the United Kingdom, and Canada. The above three aspects indicate that the United States is the most influential country.

**Table 1:** The top 10 productive countries/regions.

Rank	Country	Documents	Citation
1	United States	1,089	57,054
2	China	860	18,269
3	Germany	283	17,237
4	Brazil	223	3,963
5	United Kingdom	193	14,056
6	Japan	173	5,960
7	Italy	164	5,672
8	India	148	3,823
9	South Korea	147	3,310
10	Canada	129	4,471

### Distribution of source journals and 10 most cited articles

A total of 3,894 papers on skin inflammation and regeneration were published in 1,184 journals. Table 2 lists the top 10 journals with the most published literature, accounting for 16.2% of the literature (630/3,894). *Wound Repair and Regeneration* was found to be the most productive journal (111 papers), and *Journal of Investigative Dermatology* was found to be the most cited journal (8,749 citations). Furthermore, although only three papers were published in *Physiological Reviews*, they have been cited 3,470 times in total.

**Table 2:** The top 10 journals for publications.

Rank	Journals	Documents	Citation	Category	IF (2022)
1	*Wound Repair and Regeneration*	111	4,595	Dermatology	3.401
2	*Journal of Investigative Dermatology*	93	8,749	Dermatology	7.590
3	*International Journal of Molecular Sciences*	86	2,212	Biochemistry and molecular biology	6.208
4	*PLoS One*	67	1,991	Multidisciplinary	3.752
5	*Experimental Dermatology*	53	1,899	Dermatology	4.511
6	*Scientific Reports*	51	1,280	Multidisciplinary sciences	4.996
7	*Journal of Ethnopharmacology*	51	1,556	Integrative and complementary medicine	5.195
8	*International Wound Journal*	41	1,075	Dermatology	3.099
9	*Advances in Wound Care*	39	1,710	Dermatology	4.947
10	*Frontiers in Immunology*	38	618	Immunology	8.786

IF, impact factor.

Among all papers, 283 were cited over 100 times. Table 3 lists the 10 most cited papers. *Regulation of wound healing by growth factors and cytokines* published in *Physiological Reviews* in 2003 is the most cited article (2,422 citations).

**Table 3:** The top 10 highest cited articles.

Rank	Title	Journal	Citation	PY
1	Regulation of wound healing by growth factors and cytokines	*Physiological Reviews*	2,422	2003
2	Inflammation in wound repair: Molecular and cellular mechanisms	*Journal of Investigative Dermatology*	1,372	2007
3	Wound healing: An overview of acute, fibrotic and delayed healing	*Frontiers in Bioscience-Landmark*	1,342	2004
4	LYVE-1, a new homologue of the CD44 glycoprotein, is a lymph-specific receptor for hyaluronan	*Journal of Cell Biology*	1,189	1999
5	Wound Repair and Regeneration	*European Surgical Research*	948	2012
6	Inflammation and wound healing: the role of the macrophage	*Expert Reviews in Molecular Medicine*	925	2011
7	Regulation of matrix metalloproteinases: An overview	*Molecular and Cellular Biochemistry*	915	2003
8	Keratinocyte-fibroblast interactions in wound healing	*Journal of Investigative Dermatology*	830	2007
9	Cathelicidins, multifunctional peptides of the innate immunity	*Journal of Leukocyte Biology*	776	2014
10	Functions of hyaluronan in wound repair	*Wound Repair and Regeneration*	764	1999

PY, published year.

### Distribution and co-authorship of institutions

A total of 4,123 institutions have conducted research on skin inflammation and regeneration. Table 4 lists the top 10 institutions with the largest number of publications. Harvard University (43 papers, 3,916 citations) was found to be the most influential institution in this field, with 43 papers and the highest number of citations. Although Shanghai Jiao Tong University has published numerous papers, the number of citations is relatively low (1,621 citations).

**Table 4:** The top 10 productive institutions.

Rank	Organization	Country	Documents	Citation
1	Shanghai Jiao Tong University	China	63	1,621
2	The University of Manchester	England	46	2,391
3	The Ohio State University	United States	43	2,864
4	Harvard University	United States	43	3,916
5	University of Illinois	United States	37	3,745
6	University of Sao Paulo	Brazil	36	777
7	Chinese Academy of Sciences	China	34	768
8	University of Miami	United States	34	966
9	University of California, Davis	United States	32	1,317
10	University of Pennsylvania	United States	32	1,871

**Figure 4:**
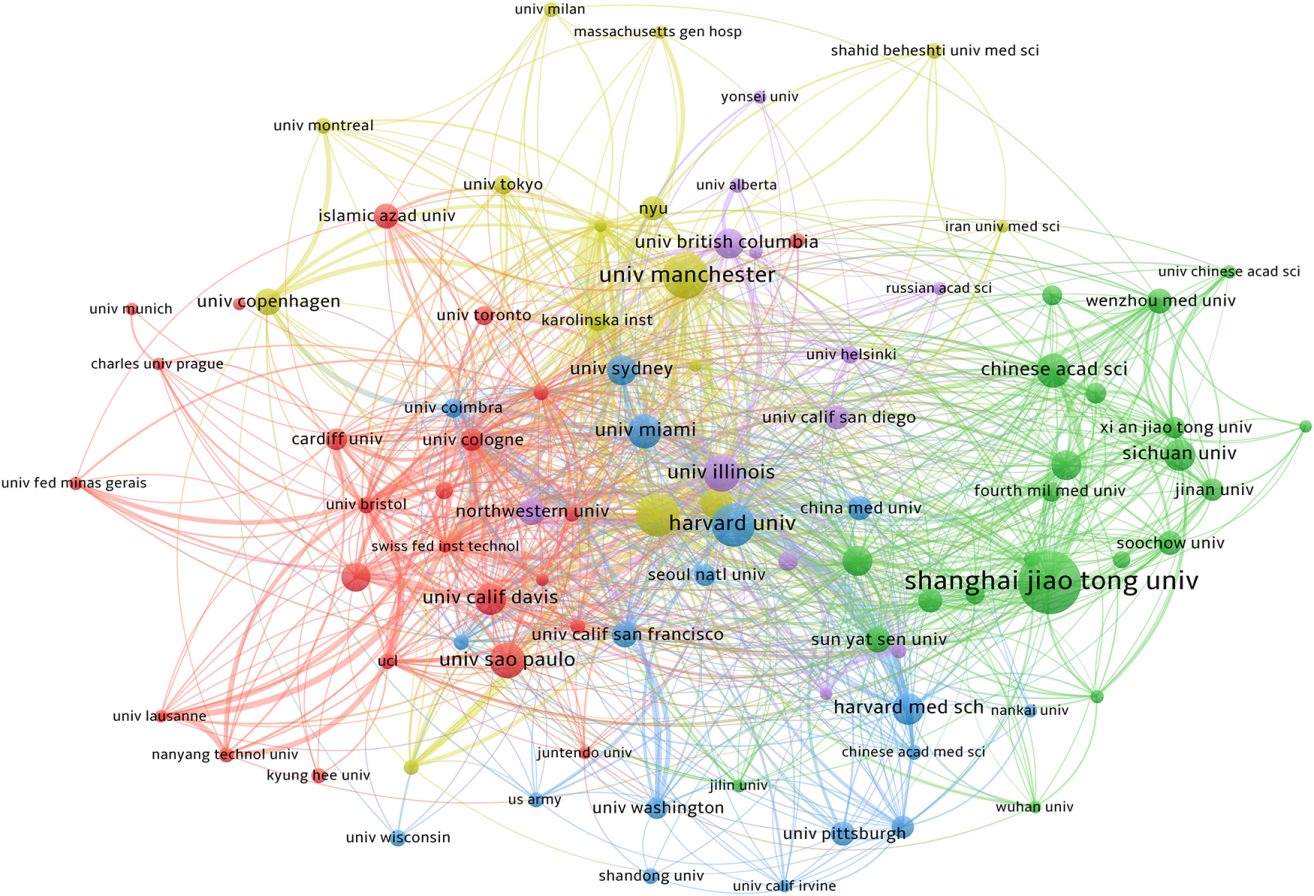
The co-authorship network of institutions. A total of 4,123 institutions participated in the study. The network of co-authors was divided into five clusters of different colors. The red cluster is dominated by American institutions, and the Harvard University has the greatest impact. The green cluster is dominated by Chinese institutions.

The cooperation network of the research institutions is shown in Figure 4. After reviewing institutions with over 10 published papers, we divided the network of co-authors into five color-coded groups, of which the largest two were green and red. The red group consists of 59 universities focused on The University of Manchester, University of Illinois, and Ohio State University. In both the green and red groups, most cooperative relations are limited to China. For example, the universities in the green group are Chinese.

### Distribution and co-authorship of authors

**Figure 5:**
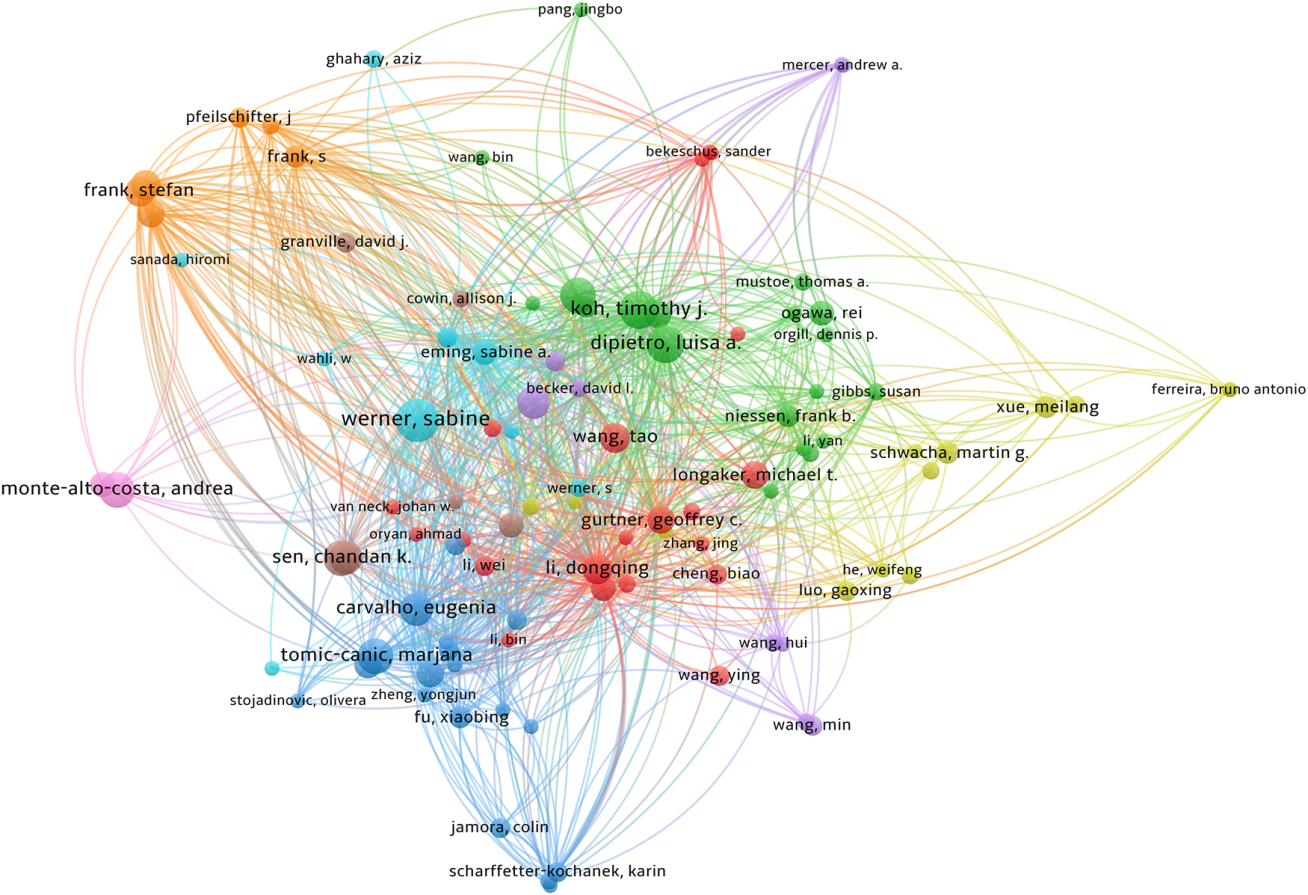
The co-authorship network of authors. A total of 21,144 authors participated in the writing process. The analysis method is Lin Log/modular. The size of the circle represents the influence of the author. Internode connections represent author collaboration and the lines of connection between nodes represent the collaboration between authors.

A total of 21,144 authors participated in the writing of the 3,894 papers. The top 10 authors with the most published papers are listed in Table 5. The table shows that Werner Sabine (17 publications, 2,076 citations) is the most prolific and cited author in this field. A threshold of six articles was set in VOSviewer, and 103 authors who met the criteria were selected. The results are shown in Figure 5. The nine color-coded groups in the figure represent the most closely linked groups in this field.

**Table 5:** The top 10 productive authors.

Rank	Author	Total publications	Citation	Avg citation
1	Werner, Sabine	17	2,076	122
2	Dipietro, Luisa A	15	1,392	92
3	Koh, Timothy J	15	1,624	108
4	Wilgus, Traci A	14	582	41
5	Sen, Chandan K	14	694	49
6	Tomic-Canic, Mariana	14	469	33
7	Monte-Alto-Costa	14	331	23
8	Andrea Carvalho	13	687	52
9	Eugenia Martin, Paul	13	1,351	104
10	Roy, Sashwati	13	684	53

### Co-citation analysis of cited references

**Figure 6:**
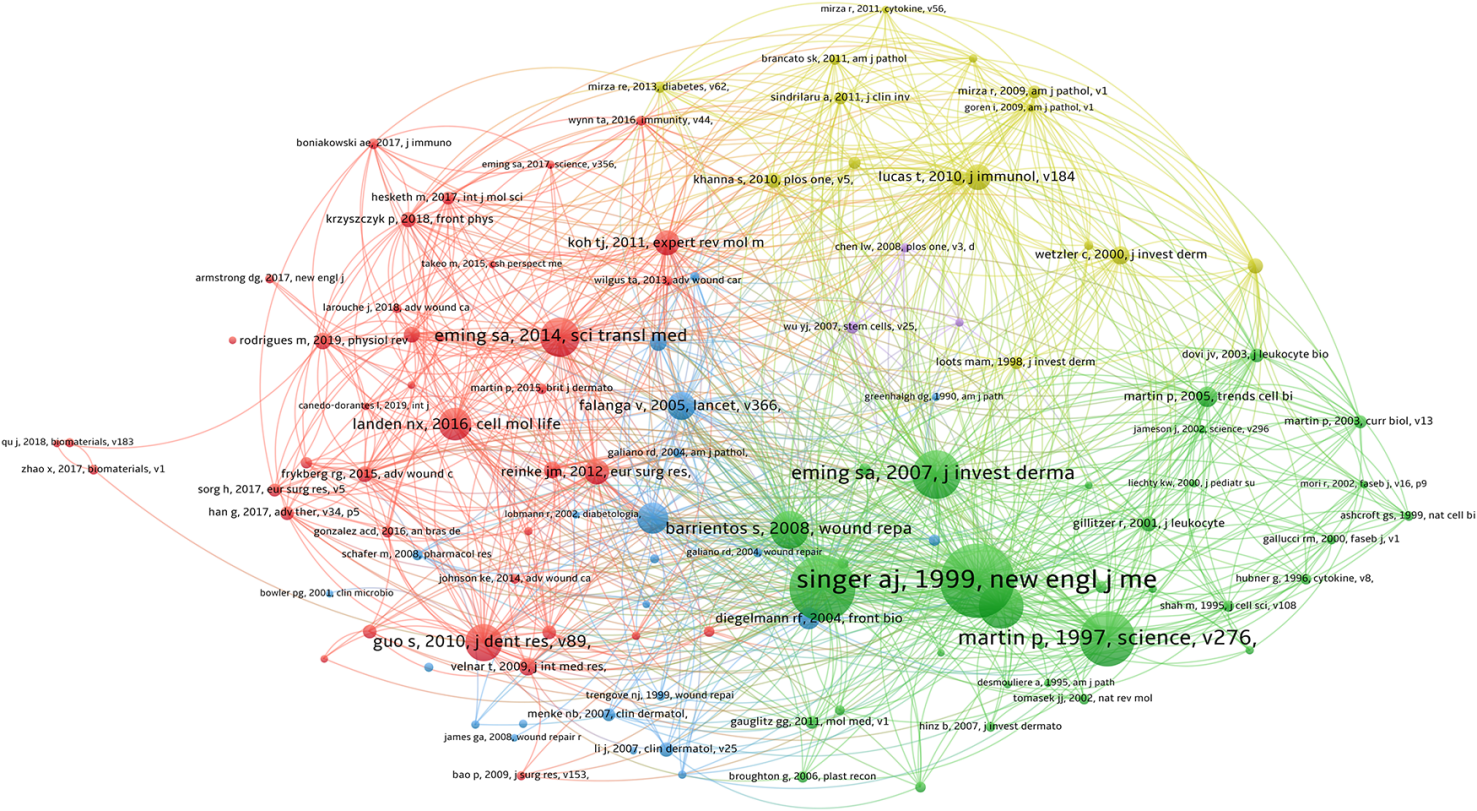
Co-citation analysis of references. The most cited article in co-citation analysis were written by A. J. Singer. Clusters of the same color indicate that the literature studies are related or have some commonalities.

Following paper co-citation analysis, the 10 most cited papers were selected and are listed in Table 6, which is visualized in Figure 6. The paper titled *Mechanisms of disease-Cutaneous wound healing* published in the *New England Journal of Medicine* in 1999 was found to be the most cited (391 citations).

**Table 6:** The top 10 most co-cited references.

Rank	First author	Year	Journal	Title	Citations
1	Singer, AJ	1999	*New England Journal of Medicine*	Mechanisms of disease—Cutaneous wound healing	391
2	Gurtner, Geoffrey C	2008	*Nature*	Wound repair and regeneration	348
3	Martin, P	1997	*Science*	Wound healing–aiming for perfect skin regeneration	291
4	Eming, Sabine A	2007	*Journal of Investigative Dermatology*	Inflammation in wound repair: Molecular and cellular mechanisms	256
5	Werner, S	2003	*Physiological Reviews*	Regulation of wound healing by growth factors and cytokines	237
6	Eming, Sabine A	2014	*Science Translational Medicine*	Wound repair and regeneration: Mechanisms, signaling, and translation	211
7	Barrientos, Stephan	2008	*Wound Repair and Regeneration*	Growth factors and cytokines in wound healing	200
8	Guo, S.	2010	*Journal of Dental Research*	Factors Affecting Wound Healing	196
9	Landen, Ning Xu	2016	*Cellular and Molecular Life Sciences*	Transition from inflammation to proliferation: a critical step during wound healing	175
10	Sen, Chandan K	2009	*Wound Repair and Regeneration*	Human skin wounds: A major and snowballing threat to public health and the economy	167

**Figure 7:**
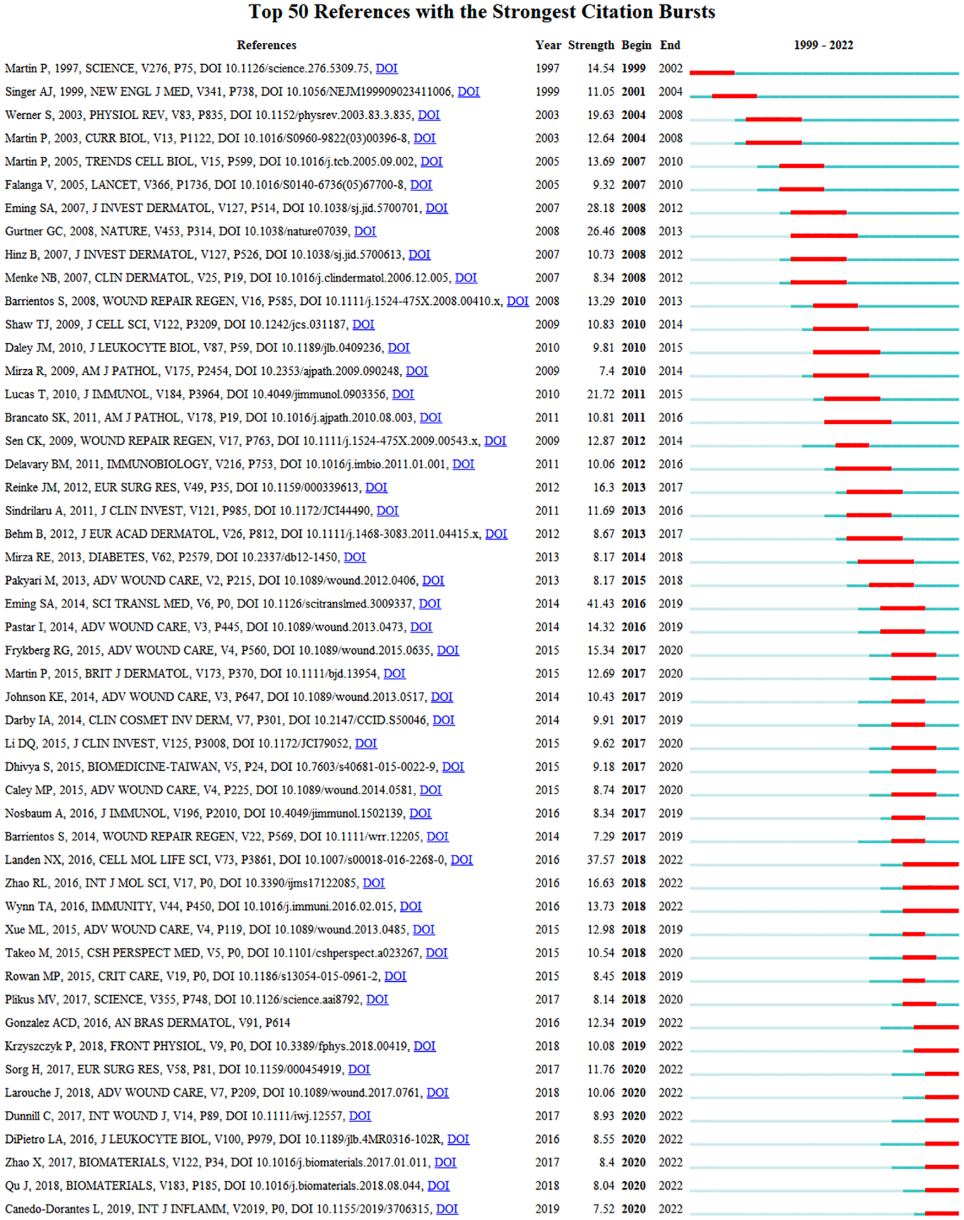
CiteSpace visualization map of top 50 references with the strongest citation bursts. A citation burst is when the citation situation of a paper changes dramatically in a short period of time. The red line indicates the duration of the outbreak, and the intensity of the outbreak indicates the impact of the article. The citation burst is analyzed with CiteSpace.

A strong citation burst analysis can identify published papers that have experienced significant changes in their citation situation over a short time period. The red line indicates the duration of the burst, and the intensity of the burst is indicative of the influence of the published paper. Figure 7 shows the first 50 papers with the strongest citation bursts. *Wound repair and regeneration: Mechanisms, signaling, and translation* published in *Science Translational Medicine* in 2014 had the highest burst intensity of 41.43. The high-intensity citation burst in this study lasted until 2019. This review article reviews and summarizes recent advances in skin regeneration and presented some beneficial perspectives on promoting wound healing in clinical patients.

### Co-occurrence analysis of the top 50 keywords

Keywords summarize the theme and content of the published paper. The frequency analysis of keywords is the most important window for understanding research hotspots and trends. We used VOSviewer to analyze the top 50 keywords (Table 7). After excluding keywords with vague directivity, such as sound healing and skin, we found that the following keywords appeared frequently: angiogenesis (166), diamonds (105), collagen (90), cytokines (76), and keratinocytes (70).

**Table 7:** The top 50 keywords.

Keywords	Counts	Rank	Keywords	Counts	Rank
Wound healing	1,295	1	Mesenchymal stem cells	46	26
Inflammation	607	2	Fibroblast	44	27
Skin	269	3	Anti-inflammation	43	28
Angiogenesis	166	4	Proliferation	42	29
Diabetes	105	5	Diabetes mellitus	41	30
Collagen	90	6	Burn	40	31
Wound	82	7	Diabetic wound healing	39	32
Cytokines	76	8	Antibacterial	39	33
Keratinocytes	70	9	Tissue regeneration	38	34
Wound dressing	65	10	Tissue engineering	38	35
Chronic wounds	63	11	Regeneration	38	36
Anti-inflammatory	62	12	Chronic wound	38	37
Fibrosis	60	13	Verge	35	38
Hydrogel	59	14	Hyaluronic acid	35	39
Fibroblasts	57	15	Diabetic foot ulcer	34	40
Macrophage	54	16	Exosomes	34	41
Oxidative stress	53	17	Scar	33	42
Healing	53	18	Diabetic wound	33	43
Macrophages	51	19	Nitric oxide	32	44
Skin regeneration	50	20	Infection	32	45
Extracellular matrix	50	21	Tissue repair	31	46
Antioxidant	48	22	Re-epithelialization	31	47
Skin wound healing	47	23	Hypertrophic scar	31	48
Keratinocyte	47	24	Electrospinning	31	49
Chitosan	47	25	Growth factors	30	50

**Figure 8:**
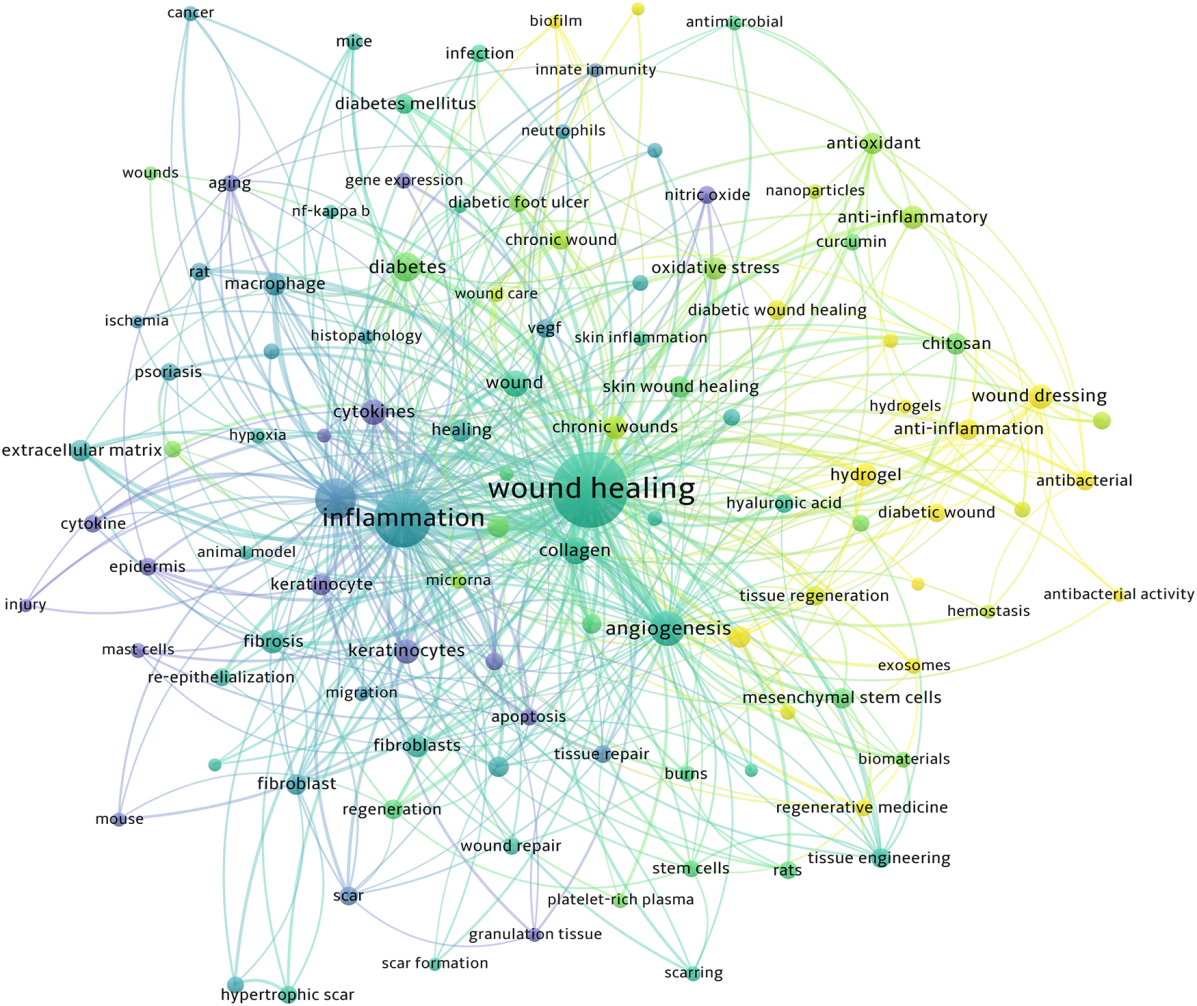
The overlay map of keywords. A node's size indicates how often the keyword appears. Cooler colors indicate earlier occurrences of keywords, and warmer colors indicate later occurrences.

In the Overlay visualization view of VOSviewer (Figure 8), the keywords are colored according to their average occurrence year (AAY). A cooler color indicates that the keywords appeared earlier, while a warmer color indicates that they appeared later. The keywords that recently attracted attention were exosomes (AAY: 2021), hydrogel (AAY: 2020), and wound dressing (AAY: 2019).

**Figure 9:**
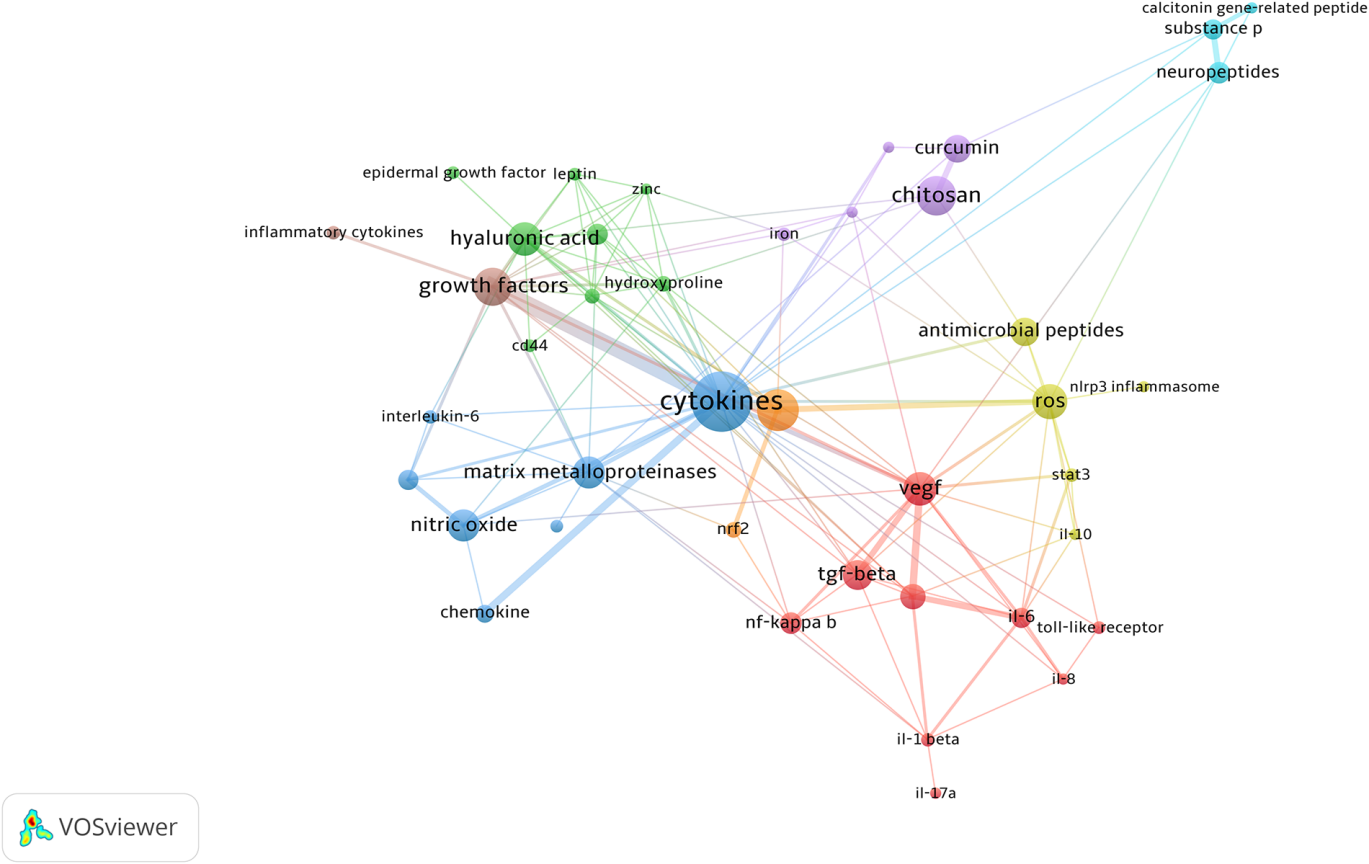
The keywords co-occurrence network visualization. High-frequency keywords with high correlation are shown with the same color, for example, high-frequency keywords of cytokines such as VEGF, TNF-α, and interleukin are shown in red.

After selecting keywords related to regulatory molecules and pathways, we listed the top 20 keywords with the most frequent occurrence in Table 8, and then visualized them with VOSviewer (Figure 9). The keywords that have appeared most frequently are cytokines; many high-frequency keywords in the table belong to this category, such as VEGF, TGF-*β*, and TNF-α. Cytokines play an important role in all stages of wound healing. Therefore, studies on the effects of cytokines, growth factors, and downstream effectors in wound healing have always been a hot topic in this field.

**Table 8:** The top 20 keywords.

Keywords	Counts	Rank	Keywords	Counts	Rank
Cytokines	105	1	Chemokines	27	11
Oxidative stress	53	2	Antimicrobial peptides	26	12
Chitosan	47	3	Curcumin	25	13
Growth factors	44	4	TNF-α	21	14
Ros	38	5	Neuropeptides	16	15
Hyaluronic acid	35	6	NF-*κ*b	16	16
VEGF	35	7	Insulin	15	17
Matrix metalloproteinases	33	8	Cyclooxygenase	14	18
Nitric oxide	32	9	IL-6	14	19
TGF-β	29	10	Substance p	14	20

## Discussion

In this study, we used bibliometrics to analyze the current research hotspots and future research trends in the field of skin inflammation and regeneration and found that the number of publications on skin inflammation and regeneration showed an increasing trend over time. Additionally, authors and institutions in the United States, United Kingdom, Canada, and China are at the forefront of research in the field of skin inflammation and regeneration. Finally, a series of advanced research results and treatments for skin inflammation and regeneration are emerging, such as the discovery of key regulatory mechanisms of inflammation, which will provide precise strategies for skin regeneration.

In this study, 3,894 papers on skin inflammation and healing published between January 1, 1999, and December 31, 2022, were searched on WOS. Overall, the evolution of published figures over time is indicative of the importance and progress of related research in the field of skin inflammation and regeneration. Currently, the United States, China, and Germany are the top three countries with the highest number of publications. Citation bursts are focused on the United States, the United Kingdom, and Canada, indicating that the United States is the most influential country. Additionally, Shanghai Jiao Tong University in China, the University of Manchester in England, Ohio State University, Harvard University, and University of Illinois in the United States published the highest number of papers. Harvard University became the most influential institution in this field with 43 papers and the largest number of citations. In terms of global journal quality, the highest number of papers on skin inflammation and regeneration were published in *Wound Repair and Regeneration*. *Frontiers in Immunology* is the journal with the highest impact factor (IF), IF 2021 = 8.786.

Dr. Werner of ETH Zurich has published 17 papers with the most citations, ranking himself as the leading author in the field of skin inflammation and regeneration. Dr. Werner in 2003 published one review “*Regulation of wound healing by growth factors and cytokines*” (28) on *Physiological Reviews*, which is the most cited paper with 2,422 citations. This review indicates that skin healing is a complex process that involves inflammation and the formation of new tissues. It summarizes numerous approaches focused on growth factors, cytokines, and their receptors or downstream effectors in skin repair. The co-authorship map indicated an increasing pattern of cooperation among researchers interested in skin regeneration. The most cited research is by Dr. Quigley, from Johns Hopkins Hospital, and “*Mechanisms of disease: Cutaneous wound healing*” (29) published in *New England Journal of Medicine*, which has been cited 391 times. This article discusses the biology and the role of skin substitutes in wound regeneration. It also indicates that macrophages appear to facilitate wound repair. Furthermore, “*Wound repair and regeneration: Mechanisms, signaling, and translation*” (30) published in *Science Translational Medicine* in 2014 has the highest burst intensity of 41.43. This review article reviews and summarizes recent advances in skin regeneration and presents some beneficial perspectives on the promotion of wound healing in clinical patients.

Co-occurrence group analysis was used to analyze the network of keywords studied in skin inflammation and regeneration research. Current research hotspots can be identified based on the keyword frequency. Some of the 50 most common keywords were oxidative stress, collagen, cytokines, and exosomes (31–34). The keyword results showed that the research trend of skin inflammation and regeneration is diversifying, which is related to cell biology and biochemistry (35, 36). Furthermore, analysis of frequency and centrality of keywords revealed that treatments such as “wound dressing,” “tissue engineering,” and “chitosan” appeared earlier (37–39), suggesting that further research on treatment methods is needed. Additionally, keywords such as “exosomes” and “hydrogel” began to attract attention mainly after 2016, suggesting that this research area is the next research direction and hotspot (40, 41).

Numerous studies have indicated that inflammation is an important differentiating factor in wound repair processes (42, 43). Analyzing the key molecules in skin regeneration and inflammation revealed that the frequency of some keywords increased rapidly in recent years. We analyzed and discussed the most commonly used keywords. For example, dendritic epidermal T cells can promote epithelial proliferation by producing keratinocyte growth factor-2 and IL-6, which induce signal transducer and activator of transcription 3 (44, 45). Additionally, damaged epithelial cells secrete alarmin IL-18, which activates resident Tc17 cells to produce the transcription factors GATA3 and IL-13, ultimately promoting wound regeneration (46–48). Furthermore, aberrant inflammation also leads to impaired wound healing, such as wound Tregs curbing Th17 responses that hinder skin healing (49, 50).

## Limitation

In this study, which was completed on March 1, 2023, we collected papers published between January 1, 1999, and December 31, 2022. Consequently, some previously and subsequently published papers with updated results may not be included, making the papers in our database fewer than practical papers. In addition, only articles and reviews were included in this research, as we wanted to control the quality of publications to the maximum possible extent. Therefore, other types of papers might have been overlooked, such as meta-analyses, books, and case reports, which may have advanced achievements in both fundamental and clinical research. Finally, the field of skin inflammation and regeneration covers numerous aspects of the research. Although we retrieved the most relevant publications, some important research results may have been neglected due to the limitations of the bibliometric tools used.

## Conclusion

The purpose of this study was to summarize the current research hotspots and future trends in skin inflammation and regeneration. We used bibliometrics to analyze related publications in the field and found that publications on skin inflammation and regeneration show an increasing trend. Furthermore, a series of advanced research results, technology, and treatment for skin regeneration are emerging, such as the research findings of key molecules and mechanisms of inflammation and immunity, which will provide precise information for the treatment of skin injuries. Taken together, this research can enhance our understanding of the current hotspots and future trends in the field of skin inflammation and regeneration and will provide guidelines for fundamental research and clinical treatment.

## Data Availability

The original contributions presented in the study are included in the article/Supplementary Material, further inquiries can be directed to the corresponding authors.
